# Defining the Stem Cell Niche in Developing Pericoronal Dental Follicles

**DOI:** 10.7759/cureus.93712

**Published:** 2025-10-02

**Authors:** Zornitsa Mihaylova, Marina Miteva, Maria Praskova, Silvia Kalenderova, Violeta Dimitrova, Evgeniy Aleksiev, Riana Cockeran, Nikolay Ishkitiev, Vanyo Mitev

**Affiliations:** 1 Research Institute of Innovative Medical Science, Medical University of Sofia, Sofia, BGR; 2 Department of Dental, Oral, and Maxillofacial Surgery, Medical University of Sofia, Sofia, BGR; 3 Department of Medical Chemistry and Biochemistry, Medical University of Sofia, Sofia, BGR; 4 Department of Cellular Therapy and Novel Products, South African National Blood Service, Johannesburg, ZAF

**Keywords:** dental follicle stem cells, mesenchymal stem cells (mscs), pericoronal follicular tissue, stem cell markers, stem cell research

## Abstract

Objective: The pericoronal dental follicle is a connective tissue structure encased in epithelium present around the developing tooth germ before erupting and emerging in the oral cavity. Stem cells from pericoronal follicles can be obtained and cultured under suitable conditions in vitro. This study builds upon our previous research, with the primary objective of identifying stem cell subpopulations in the oral cavity based on marker expression and differentiation potential.

Materials and methods: Dental follicle stem cells (DFSCs) were obtained and cultured under standard cell culturing conditions. Cellular phenotype and the expression of stem cell and tissue markers were analyzed using high-throughput fluorescent analysis. Additionally, the cells were cultured for 10 days in differentiation media to assess their osteogenic, adipogenic, and chondrogenic differentiation potential in vitro. Stem cell differentiation was evaluated using Alcian Blue, Oil Red O, and Alizarin Red staining.

Results: The stem cells isolated by the research team showed positive fluorescent expression for epithelial markers and mesenchymal markers (CD44H, CD71, CD90). High-content cellular analysis enabled the quantitative profiling of DFSC marker expression. Under appropriate conditions, DFSCs demonstrated the ability to differentiate into osteoblast-like, chondroblast-like, and adipocyte-like cells in vitro.

Conclusion: The dental follicle contains a population of stem cells with a specific phenotype capable of multilineage differentiation. It may represent another promising source of human adult stem cells. The observed differentiation profile may limit the application of the isolated cells in the hard tissue regeneration and suggest a potentially more suitable role in soft tissue engineering or immunomodulatory therapies.

## Introduction

The dental follicle (DF) is an ectomesenchymal structure located around the developing tooth prior to eruption and plays a crucial role in tooth morphogenesis. It originates from neural crest-derived mesenchyme and contains undifferentiated progenitor cells that contribute to the formation of the tooth root, periodontal ligament, cementum, and alveolar bone [1,2].

DF tissues give rise to multiple cell types involved in periodontal tissue development, including cells of the periodontal ligament and dental cementum, and are closely related to the reduced enamel epithelium, a bi-layered structure formed after enamel deposition through the collapse of the inner and outer enamel epithelium [3].

The presence of precursor cells in the human pericoronal follicular tissue was first described by Morsczeck et al. in 2005, identifying them as dental follicle stem cells (DFSCs) [4]. These cells exhibit fibroblast-like morphology, colony-forming ability, plastic adherence, and expression of putative stem cell markers such as Notch-1 and nestin. Under specific in vitro conditions, DFSCs are capable of differentiating into calcified tissues, although their osteogenic differentiation appears less complete and the underlying mechanisms remain unclear [5].

DFSCs have attracted increasing attention as a promising source of mesenchymal stem cells due to their accessibility and the relatively larger volume of residual follicular tissue compared to other dental sources, such as dental pulp or periodontal ligament [4]. Furthermore, the odontogenic tissue surrounding unerupted teeth has demonstrated potential for differentiation into various tissue types, including cystic and even neoplastic tissues [6-8].

Almost all DFs are partially lined by epithelium that is frequently fragmented or separated from the connective tissue [7], which may reflect the dynamic remodeling processes taking place during tooth eruption and development.

In our previous studies, we have isolated and characterized stem cell cultures from several oral sources, including adult and deciduous dental pulp (dental pulp stem cells (DPSC) and stem cells from human exfoliated deciduous teeth (SHED)), apical papilla (stem cells from the apical papilla (SCAP)), and periodontal ligament (periodontal ligament stem cells (PDLSC)), and compared them with gingival fibroblasts (GF) and bone marrow-derived cells (BMC) [9-11]. We have also investigated the effects of various growth factors on the stem cell properties of these populations [12].

The present study aims to characterize the biological and phenotypic properties of DFSCs, a population that has been relatively overlooked in comparative studies of oral-derived stem cells. By including DFSCs, we seek to complete the profiling of all major subpopulations of mesenchymal stem cells of oral origin. This research builds upon our previous work and is supported by our long-term experience with stem cell isolation and characterization methodologies [12].

## Materials and methods

The study was approved by the Medical Science Council of the Medical University of Sofia (approval number: 4770/11.12.2018). Cells were obtained from DFs of humans younger than 25 years (16-18 years old; n=5) without systemic diseases and healthy oral status, who had signed informed consent [12]. The inclusion criteria for this research required intact teeth without inflammation or caries infiltration. These teeth are typically extracted as part of orthodontic treatment and may contribute to complications in occlusal development. Teeth were excluded from the study if they were fully erupted or showed signs of infection, like pulpitis, periodontitis, or periapical infection. All patients (or their guardians) participating in the study signed an informed consent form prior to sample extraction [12]. 

Cell isolation and cultivation 

Immediately after extraction, the teeth were stored in cell culture medium or sterile saline. DFs were separated from the calcified dental structures and washed three times with phosphate-buffered saline (PBS) (Lonza, Verviers, Belgium). Tissue explants were sliced into small pieces (nearly 1-3 mm^2^) with sterile scalpel blades and enzymatically digested in a solution of 3 mg/ml collagenase type I and 4 mg/ml dispase (Sigma-Aldrich, St. Louis, Missouri, United States) for 15-30 minutes at 37°C and then transferred to 2 cm^2^ culture dishes with Dulbecco's Modified Eagle Medium (DMEM) (Invitrogen, Eugene, Oregon, United States) supplemented with 1% antibiotic-antimycotic (Invitrogen) and 10% heat inactivated fetal bovine serum (FBS) (Sigma-Aldrich). Cell cultures were incubated at 37°C in a humidified atmosphere of 5% CO2 and 95% air for a period of 2-4 weeks. Cells between the third and fifth passages were used in all experiments.

Characterization of DF cell cultures

DF cells were characterized for the expression of surface and intracellular markers via immunocytochemistry and flow cytometry. All experiments were carried out at least three times.

Antibodies used

Cells were incubated with the following non-conjugated antibodies: mouse anti-human alkaline phosphatase (ALP) (Sigma-Aldrich); mouse anti-human CD117, nanog, Bcl-2, COL1a2, COL3a1, vimentin, nestin, CD44H, DSPP, and Oct3/4 (all Santa Cruz, Santa Cruz, California, United States); mouse anti-human E-cadherin, CK10, and Int α2β1 (all Abcam, Cambridge, United Kingdom); mouse anti-human BMP4 and FGF7 (both RnD Systems, Minneapolis, Minnesota, United States); and rabbit anti-human CK19 and p63 N-cadherin (both Abcam). CFL488-conjugated goat anti-mouse and CFL555-conjugated goat anti-mouse, CFL488-conjugated goat anti-rabbit, and CFL555-conjugated mouse anti-rabbit (all Santa Cruz) were used as secondary antibodies (1:1000). Cells were also incubated with the following conjugated antibodies: phycoerythrin (PE)-conjugated rat anti-human CD49f (Thermo Fisher Scientific Inc., Waltham, Massachusetts, United States), allophycocyanin (APC)-conjugated mouse anti-human CD271 (Miltenyi Biotec, Bergisch Gladbach, North Rhine-Westphalia, Germany), PE-conjugated mouse anti-human CD90 (Beckman Coulter International SA, Brea, California, United States), fluorescein isothiocyanate (FITC)-conjugated mouse anti-human CD71 (Beckman Coulter), and Alexa Fluor 568-conjugated rabbit anti-human Sox9 (Abcam) at dilutions 1:1000.

Flow cytometry

When the cultures reached sub-confluence, the cells were trypsinized, washed with PBS, and exposed to bovine serum albumin (BSA)-PBS 2% for 15 minutes. For the purpose of this study, only the expression of surface markers was determined by flow cytometry; therefore, a permeabilization step was not required.

Saturating levels of conjugated or non-conjugated antibodies (1:250) were used for 30 minutes at room temperature. CFL488-conjugated goat anti-mouse antibody (Santa Cruz) (1:1000) was used as a secondary antibody for 20 minutes in the dark. Unstained cells were used as negative controls. A minimum of 10,000 cells of each cell type per individual antibody staining were analyzed on the Navios flow cytometer (Beckman Coulter).

Immunofluorescence 

Cell cultures were observed with two different instruments: the first one is a confocal scanning laser fluorescence microscope (Leica Microsystems GmbH, Wetzlar, Germany), and the second one is an IN Cell Analyzer 6000 imaging system-laser confocal slit system (GE Healthcare, Pittsburgh, Pennsylvania, United States).

The DFSCs for high-content cellular analysis were incubated in 96-well plates. The IN Cell Analyzer 6000 imaging system enabled us to identify and quantify the cell marker expression. We analyzed the entire plates with two-channel color recording. The first channel (blue) identified fluorescently stained nuclei; the other channel (green) recorded the specific fluorescent protein used for receptor tracking. A total of 36 fields of view per well were photographed at ×20 magnification, and cells were analyzed with IN Cell Analyzer work station 3.7.3 (×64) (GE Healthcare). Values in the cell channel for cell fluorescence/background fluorescence were collected, and percentages of positive cells were calculated (see Appendices).

Criteria for positivity

All values ≥1.05 and <3 were considered positive. Values <1.05 were negative (as determined with unstained cells), and values ≥3 were excluded due to autofluorescence. The threshold values used in high-content cellular analysis (e.g., ≥1.05 as positive and ≥3 as autofluorescence exclusion) were established based on the analysis of unstained controls and background fluorescence.

In vitro differentiation

In vitro cell differentiation of DF cell cultures was induced using previously described methods [13] by osteogenic, chondrogenic, and adipogenic cell culture media.

Statistical analysis

High-content cellular analysis was performed on data collected from 6000 cells stained with each marker, using IBM SPSS Statistics for Windows, Version 23.0 (IBM Corp., Armonk, New York, United States). Differences between stained and unstained cells were assessed using the Mann-Whitney U-test (non-parametric, unpaired), with p<0.05 considered statistically significant.

## Results

DFSC cultures

**Figure 1:**
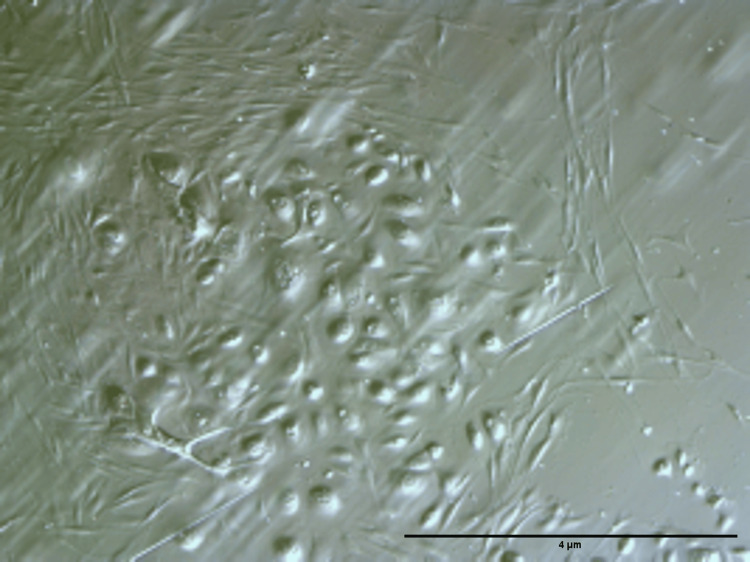
Isolated cells from the dental follicle under phase contrast light microscopy (magnification ×10). Phase contrast microscopy of DFSC mixed cultures showed plastic-adherent cells with fibroblast-like spindle morphology forming colonies, alongside a minority of rounded epithelial-like cells. The cultures reached 80% confluence within 10-21 days, and over 2-3 weeks, mesenchymal fibroblast-shaped cells became clearly separated from epithelial-like cells. These observations suggest the presence of both mesenchymal and epithelial stem cell populations in human pericoronal follicular tissues DFSC: dental follicle stem cell

DFSC mixed cultures established from five different donors were successfully cultivated at standard cell culture conditions. Cells showed their plastic adherence and colony-forming ability, reaching 80% confluence by 10-21 days after seeding. Under a phase contrast microscope, various cells in the mixed culture display fibroblast-like spindle-shaped morphology. After 3-5 days of incubation, cells began to proliferate more rapidly and form colonies. In addition to the fibroblast-shaped cells, we found the presence of cells with a rounded, epithelial cell-like shape when observed microscopically (Figure 1). We suggest that there is a pool of epithelial stem cells in human pericoronal follicular tissues of dental origin. However, these cells require cultivation in specific cell culture conditions, as their culture is a matter of further research. Later on, after plating for 2-3 weeks, we observed remarkable separation of the mesenchymal fibroblast-shaped cells from those having an epithelial appearance.

Flow cytometry

**Figure 2:**
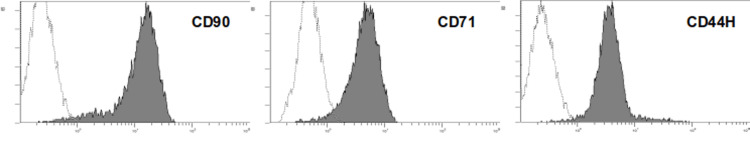
Flow cytometry histograms of the expression of stem cell markers. Flow cytometry analysis of DFSCs showed the high expression of stem cell markers CD44H, CD71, and CD90, indicating the presence of undifferentiated mesenchymal stem cells. The fluorescence profiles confirm that the majority of cells in the heterogeneous cultures retain stem cell properties. These results are consistent with the phenotype of other dental and bone marrow-derived mesenchymal stem cells DFSCs: dental follicle stem cells

Defined markers exist for the specific characterization and identification of mesenchymal stem cells in heterogeneous cell cultures. The fluorescence profile of cells was determined following immunostaining with the following markers: CD44H, CD71, and CD90. Ten thousand events per sample were analyzed, as we were able to detect the number of cells expressing the selected markers [14]. High percentages of the cells in our cultures positively express all three markers. The chosen markers were expressed by the cells, demonstrating the stem cell properties of DFSC (Figure 2). These findings are consistent with the undifferentiated state of the isolated cells and are similar to the expression profile of other mesenchymal stem cells of dental origin [15]. The cell phenotype of DFSC was also found to be similar to that revealed in bone marrow-derived mesenchymal stem cells [16].

Immunofluorescence

**Figure 3:**
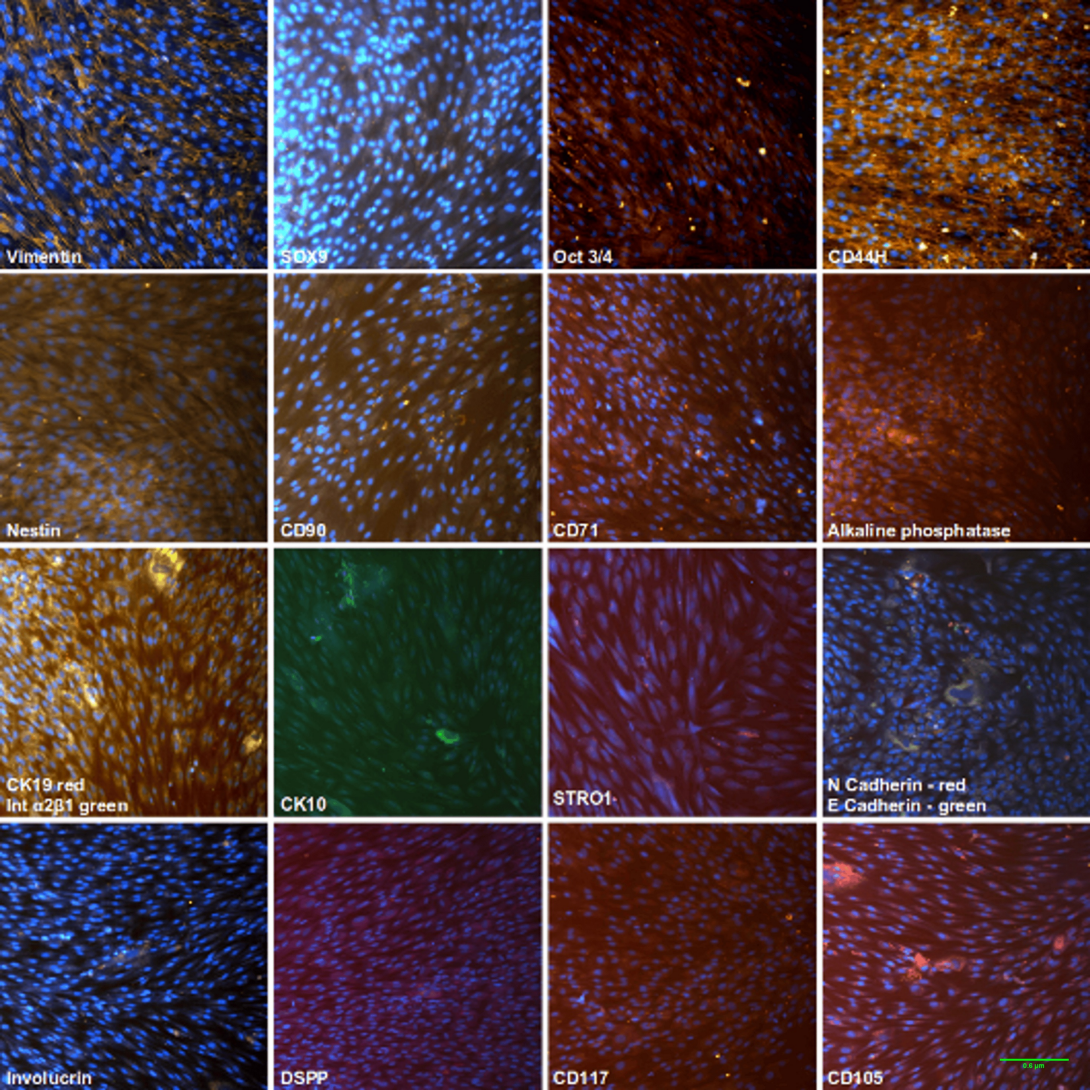
Fluorescent characterization of the expression of mesenchymal and epithelial markers (magnification ×20). Immunofluorescent staining of DFSCs showed the positive expression of stem cell markers (CD44, CD49f, CD71, CD90, CD105, vimentin, N-cadherin), epithelial markers (CK10, CK19, involucrin, integrin α2β1, E-cadherin), and ALP, confirming the presence of mesenchymal stem cells alongside epithelial-like cells in the heterogeneous culture. Counterstain: blue DAPI (4',6-diamidino-2-phenylindole) DFSCs: dental follicle stem cells; ALP: alkaline phosphatase

Immunofluorescent staining was performed to characterize the isolated DFSCs by applying a specific antibody panel for stem cell and epithelial markers. Demonstrative images are given in Figure 3. DFSC cultures show positive staining for stem cell markers (CD44, CD49f, CD71, CD90, CD105, vimentin, N-cadherin), epithelial markers (CK10 and CK19, involucrin, integrin α2β1, E-cadherin), and ALP used in the study. These findings indicate the presence of mesenchymal stem cells in the heterogeneous cell population, coexisting with epithelial-like cells (Figure 3).

High-content cellular analysis

Markers in high-throughput analysis were similarly grouped as follows: stem cell markers: CD44, CD71, CD90, Oct3/4, nestin, ALP, nanog, vimentin, CD49f, and CD271; epithelial markers: CK10, CK19, integrin α2β1, E-cadherin, FGF7, and P63; and mesenchymal differentiation markers: CD117, N-cadherin, DSPP, Sox9, BMP4, COL1a2, and COL3a1.

**Figure 4:**
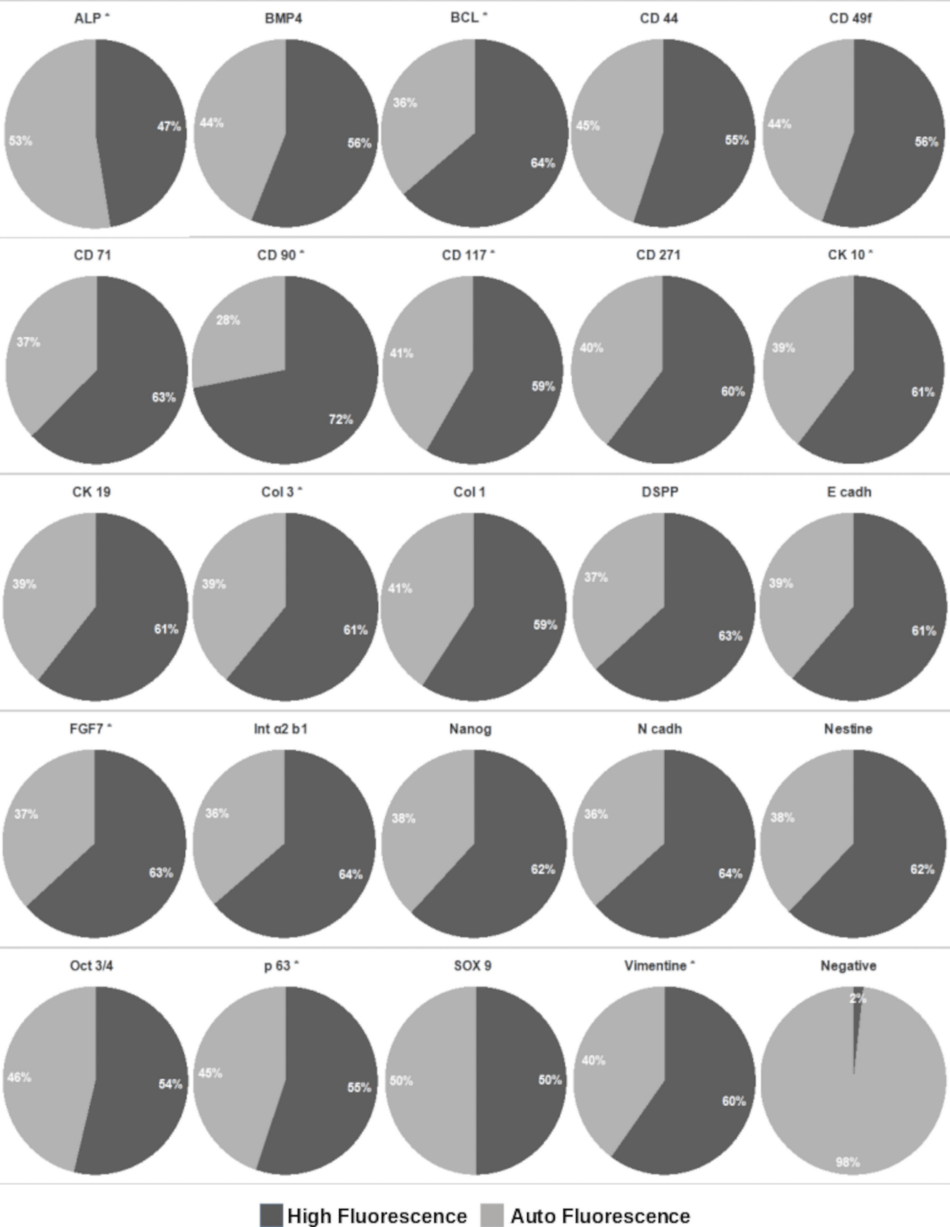
High-throughput cellular analysis of expression. The pie charts illustrate the distribution of DFSCs based on marker expression in high-throughput analysis. About one-third of cells strongly expressed stem cell markers, while CD90 was highly expressed in nearly half of the population, and multiple cells co-expressed markers from different groups. These results highlight culture heterogeneity and confirm the presence of non-differentiated stem cells alongside cells with epithelial or mesenchymal characteristics DFSCs: dental follicle stem cells

The expression of the anti-apoptotic marker BCL2 was also evaluated. Three cell subsets were identified based on marker expression intensity. Approximately one-third of the cell population strongly expressed all selected markers, indicating non-differentiated stem cells. CD90 was strongly expressed by most cells (49%). The expression of CD90, CD117, vimentin, ALP, Sox9, CK10, P63, FGF7, and COL3 was statistically significant compared to controls. Cells from each fraction could co-express markers from multiple groups, indicating culture heterogeneity (Figure 4).

In vitro cell differentiation

To identify the DFSC's ability for osteogenic, chondrogenic, and adipogenic differentiation, the cell cultures between the third and fifth passages were seeded in six-well culture plates for 21 days. At the end of the incubation period, Alizarin Red staining confirmed the presence of calcium nodules, indicating the osteogenic differentiation of the cells when cultivated in media supplemented with β-glycerophosphate, dexamethasone, and ascorbic acid.

Alcian Blue stain, capable of identifying acidic polysaccharides such as glycosaminoglycans in cartilages, was applied to verify the chondrogenic differentiation of the pericoronal DFSC. Prior to staining, the cells were cultivated in media supplemented with 100 μM ascorbic acid, 100 nM dexamethasone, as well as 5 ng/mL transforming growth factor (TGF)-β1 for three weeks' time.

**Figure 5:**
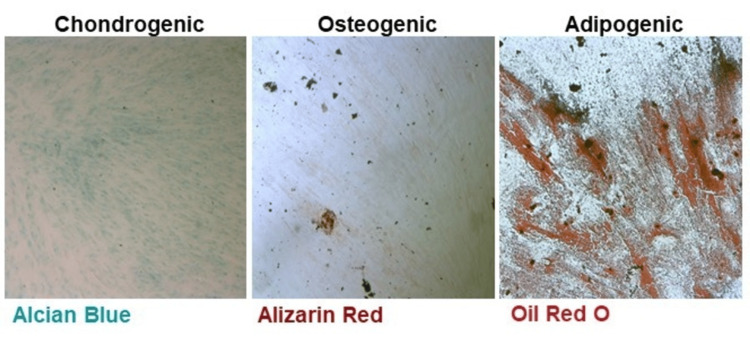
Phase contrast light microscopy after the differentiation of DFSC cultures (magnification ×5). The image demonstrates the multilineage differentiation potential of DFSCs in vitro. Osteogenic, chondrogenic, and adipogenic differentiation were confirmed by Alizarin Red, Alcian Blue, and Oil Red O staining, respectively, showing calcium nodules, glycosaminoglycan deposition, and intracellular lipid droplets. These results verify the stem cell properties of DFSCs and suggest their potential use in tissue regeneration and regenerative medicine DFSCs: dental follicle stem cells

To induce adipogenic differentiation, cells were cultivated in media supplemented with 50 μM dexamethasone, 500 nM isobutyl methyl xanthine, and 1 μg/mL insulin prior to Oil Red O staining. After 21 days of incubation, we found the presence of intracellular fat drops, revealing the stem cells' differentiation into adipocytes (Figure 5).

The successful cell differentiation into osteoblasts, chondroblasts, and adipocytes confirms the pericoronal DFSC's ability for multilineage differentiation and verifies their stem cell properties. Thus, we suggest their potential application in tissue regeneration and regenerative medicine.

## Discussion

DF is an ectomesenchymal structure surrounding the tooth germ. It plays a crucial role in the formation of the tooth crown and roots, deciduous tooth and bone resorption, and tooth eruption, as it surrounds the enamel organ and the apical papilla of the developing tooth [17]. It gives rise to cementoblasts, osteoblasts, and various types of mesenchymal stem cells, including PDLSC [18], so-called DF cells [19], or even dental follicle progenitor cells (DFPC) [20]. 

Physiologically, DFSCs remain quiescent in unerupted teeth. However, it is well known in clinical practice that these cells are capable of abnormal proliferation and may give rise to odontogenic cysts and even several types of tumors [21]. The intimate mechanisms of cystic degeneration and neoplasm formation are not yet fully elucidated, and the exact role of the undifferentiated cells remains unknown. We suspect that these cells become significantly affected by the local stimuli; thus, they no longer remain quiescent. The exact mechanism of cystic degeneration in the pericoronal DF requires further research.

The pericoronal DF is a condensed tissue that surrounds the enamel organ. All dental stem cells, including DFSCs, are derived from the neural crests and are known to have ectomesenchymal origin [22]. The neural crest comprises a pluripotent cell population that participates in odontogenesis. Neural crest cells are capable of differentiating into both ecto- and mesodermal cell lineages [23]. Therefore, it is plausible to assume that DFSCs are also able to differentiate into various germinative layer cell types.

The main goal of our study is to identify stem cell niches in human DFSC cultures and to characterize the expression of distinct stem cell markers, specific for mesenchymal and epithelial stem cells, as well as markers of differentiation. Moreover, we demonstrated the cell's ability to differentiate into various cell types when cultured in appropriate cell culture media. 

We proceeded with the investigation of DFSC properties via characterization based on the expression of specific markers. Immunofluorescence staining showed the positive expression of а panel of well-known markers in our DFSC cultures. We identified cells that expressed the mesenchymal stem cell markers CD44H, CD71, CD90, CD105, vimentin, nestin, Oct3/4, CK19, STRO1, and CD117 and also the differentiation markers Sox9, ALP, CK10, integrin α2β1, N-cadherin, E-cadherin, involucrin, and DSPP. Stem cell markers varied in intensity but were expressed throughout the entire cell populations. Differentiation markers were also expressed, although unevenly and only by single cells, which showed the heterogeneity of the isolated DFSC. The results revealed the coexistence of non-differentiated cells together with cells of ectodermal and mesodermal lineages. Vimentin and CD44H were shown to have the strongest expression compared with the other markers included in our study (Figure 2). Therefore, we may state that the majority of cells in the population are mesenchymal cells. Previous studies report the expression of most of the selected markers in the present study in other dental stem cell niches like PDLSC, DPSC, SHED, etc. [12,15,22].

Flow cytometry confirmed the expression of CD71 and CD90, together with the positive expression of CD44H. Our results are in agreement with previous reports that show the positive expression of CD90 in DFSC [19]. Studies have revealed the higher expression of CD44H in DFSCs than in bone marrow stem cells [13]. To the best of our knowledge, the present study is the first one that demonstrates the positive expression of CD71 in human DF cells. CD71 is a stem cell marker previously found in human alveolar bone mesenchymal stem cells [24]. Variability in the cell surface markers' expression might be found in the literature due to the increasing passages and the different stages of cell culture and proliferation, as well as the specific cell culture conditions [25]. A high percentage of the cells in the investigated cell cultures demonstrate the positive expression of three stem cell surface markers, which allows us to state with certainty that there is a pool of mesenchymal stem cells in the pericoronal follicular tissue. 

Further, we applied high-content cellular screening and analysis of the marker expression profile of DFSC. This allowed us to quantify the single-cell expression levels of the tested markers and to invalidate autofluorescence.

Of the tested mesenchymal markers, more than 80% of cells expressed vimentin. Nestin and Oct3/4 were positively expressed in a very limited number of cells (less than 3%), and the expression of CD271 was not statistically significant (Table 1). The high expression of vimentin confirms the mesenchymal stem cell phenotype [26].

The fibroblast-associated marker COL1a2 was expressed in less than 3% of cells, and the expression of COL3a1 was not statistically significant. FGF7 is known to promote autocrine keratinocyte growth and was also limited to less than 3% of cells. BMP4 and ALP are strongly expressed in bone and other hard tissue precursors. We found that less than 0.5% of cells expressed ALP and around 2.5% cells expressed BMP4. Finally, less than 1% of cells expressed the chondrogenic marker Sox9. We expected to see various markers' expression in the cells, as the culture is heterogeneous. However, based on the markers' expression, most of the cells reveal a typical mesenchymal stem cell phenotype. The cell culture media probably selectively stimulate the prevalence of these cells.

Although all cells exhibited the consistent expression of key stem cell markers and multilineage differentiation potential, this study does not aim to investigate inter-donor variability in stem cell properties.

According to cell phenotype in human pericoronal DF tissues and cell population heterogeneity, previous studies have also postulated the presence of epithelial-like cells. Nam and Lee [6] found positive expression for epithelial stem cell-related genes such as ABCG2, Bmi-1, DNp63, and p75 in human dental pulp cell cultures, which suggests that there is a source of epithelial cells in adult dental tissues capable of participating in tooth repair and regeneration. Currently, there is no clear evidence to confirm the presence of epithelial cells in human DF tissue. Our results demonstrate the presence of epithelial-like cells, although their long-term proliferative capacity was not established when cultivated at standard cell culture conditions. These cells have typical epithelial morphology and express specific epithelial markers. Follicular epithelial cells could play a role in the tooth regenerative events as the main epithelial component. However, we may conclude that these cells probably require specific epithelial cell-supplemented media for optimal in vitro proliferation. Further investigations are needed to elucidate their properties and applications in tissue regeneration.

In the past, various experiments have been conducted without obtaining clear evidence regarding the differentiation ability of DFSCs. It has been demonstrated that these cells have excellent proliferation ability and are capable of adipogenesis and osteogenesis [27]. Kémoun et al. [28] revealed that DFSCs are capable under appropriate conditions to differentiate towards cemento-, chondro-, and adipocytes, although another study showed only osteogenic differentiation with no ability of adipogenesis and chondrogenesis [29]. Morsczeck et al. [5] also prove the ability of DFSCs for osteogenesis. For a long period of time, these cells have been called "progenitor" cells due to the lack of sufficient data about their particular stem cell properties. Following three weeks of in vitro cultivation in cell culture media prepared according to protocols for adipogenic, chondrogenic, and osteogenic cellular differentiation, we identified significant Oil Red O labeling, confirming the lipid droplets' presence in the cellular cytoplasm. After applying chondrogenic differentiation medium, we found weak, but thorough, Alcian Blue staining of proteoglycans. Osteogenic differentiation led to limited Ca2+ nodule formation and thus weak Alizarin Red staining. The observed differentiation profile may limit the application of the isolated cells in the hard tissue regeneration and suggest a potentially more suitable role in soft tissue engineering or immunomodulatory therapies. Therefore, the relatively weak osteo- and chondrogenic differentiation may question the applicability of DFSCs in bone and cartilage regeneration, yet the cell multipotency makes them a valuable candidate for alternative regenerative therapies, i.e., periodontal ligament regenerations, soft tissue wound healing, etc.

Differentiation assays were primarily qualitative and descriptive due to the exploratory nature of the study. However, quantitative fluorescent intensity measurement was added to strengthen the analysis. More robust quantification and standardized protocols are recommended for future research. 

The presence of epithelial-like cells in heterogeneous DFSC cultures was observed, yet their precise functional role requires thorough investigation. Functional assays to elucidate their contribution and differentiation potential were beyond the scope of the current study but represent an important avenue for future research.

The study has some limitations that should be considered. The sample size was limited, which may affect the generalizability of the results. Although all samples were obtained from a narrow age range (16-18 years), this was an intentional criterion, as DFSC can only be reliably isolated from non-fully developed third molars in younger individuals. Additionally, the in vitro conditions, while informative, may not fully reflect the complexity of the in vivo environment. Nonetheless, the result provides meaningful insights into the properties and marker expression of DFSCs. Additional limitations of this study include the unconfirmed impact of passage number on marker expression, the lack of in vivo validation, and the need for further investigation into the role and function of the epithelial-like cells by the research team. 

## Conclusions

In this study, we successfully isolated and characterized DFSCs, confirming their mesenchymal origin and the presence of both mesenchymal- and epithelial-like cell populations. The DFSCs showed high expression of stem cell markers and demonstrated multilineage differentiation capacity, with the strongest potential for adipogenic differentiation under the tested conditions. These findings support the classification of DFSCs as a distinct subpopulation of oral-derived adult stem cells and highlight their potential relevance for future regenerative applications.

## Appendices

**Table 1:** Table of statistical significance of IN Cell Analyzer high-throughput cellular analysis Statistics: All data analyzed (negative, positive, and autofluorescence) One-way ANOVA, non-parametric, with Kruskal-Wallis post-test p≤0.05: significant; NS (not significant): p>0.05 DFSC: dental follicle stem cell; Nest: nestin; Vim: vimentin

DFSC	BMP4	CD44	CD49F	CD71	CD90	CD117	CD146	CD271	COL1	COL3	FGF7	Nest	Oct3/4	Sox9	Vim
ALP	<0.001	NS	<0.001	<0.001	<0.001	<0.001	<0.001	<0.001	NS	<0.001	<0.001	NS	<0.001	<0.001	<0.001
BMP4		<0.001	<0.001	<0.001	<0.001	<0.001	<0.001	<0.001	<0.001	<0.001	<0.05	<0.001	<0.001	<0.001	<0.001
CD44			<0.001	<0.001	<0.001	<0.001	<0.001	<0.001	NS	<0.001	<0.001	NS	<0.001	<0.001	<0.001
CD49F				<0.001	<0.001	<0.001	<0.001	<0.001	NS	<0.001	<0.001	<0.001	<0.001	NS	<0.001
CD71					<0.001	<0.001	<0.001	<0.001	<0.01	<0.001	<0.001	<0.001	<0.001	<0.001	<0.001
CD90						<0.001	<0.001	<0.001	<0.001	<0.001	<0.001	<0.001	<0.001	<0.001	<0.001
CD117							<0.001	NS	<0.001	<0.05	<0.001	<0.001	NS	<0.001	<0.001
CD146								<0.001	<0.001	<0.001	<0.001	<0.001	<0.001	<0.001	<0.001
CD271									<0.01	NS	<0.001	<0.001	<0.01	<0.001	<0.001
COL1										<0.05	<0.001	NS	<0.001	NS	<0.001
COL3											<0.001	<0.001	<0.001	<0.001	<0.001
FGF7												<0.001	NS	<0.001	<0.001
Nest													<0.001	<0.001	<0.001
Oct3/4														<0.001	<0.001
Sox9															<0.001
